# Transcatheter interventions in patients with a Fontan circulation: Current practice and future developments

**DOI:** 10.3389/fped.2022.965989

**Published:** 2022-08-30

**Authors:** Zakaria Jalal, Marc Gewillig, Younes Boudjemline, Patrice Guérin, Mara Pilati, Gianfranco Butera, Sophie Malekzadeh-Milani, Martina Avesani, Jean-Benoit Thambo

**Affiliations:** ^1^University Hospital of Bordeaux - Department of Pediatric and Adult Congenital Cardiology, Pessac, France; ^2^IHU LIRYC Electrophysiology and Heart Modeling Institute, Fondation Bordeaux Université, Pessac, France; ^3^INSERM, Centre de Recherche Cardio-Thoracique de Bordeaux, U1045, Pessac, France; ^4^Department of Pediatric Cardiology, University Hospitals Leuven, Leuven, Belgium; ^5^Sidra Medicine, Heart Center, Doha, Qatar; ^6^Interventional Cardiology Unit, Inserm UMR 1229, L’Institut du Thorax, University Hospital of Nantes, Nantes, France; ^7^Medical and Surgical Department of Pediatric Cardiology, Bambino Gesù Children Hospital, IRCCS, Rome, Italy; ^8^Department of Congenital and Pediatric Cardiology, Centre de Reference Malformations Cardiaques Congenitales Complexes—M3C, Necker Hospital for Sick Children, Assistance Publique des Hôpitaux de Paris, Pediatric Cardiology, Paris, France

**Keywords:** Fontan procedure, percutaneous interventions, stents, percutaneous valve, collaterals embolization

## Abstract

The Fontan operation represents the last of multiple steps that are offered a wide range of congenital cardiac lesions with a single ventricle (SV) physiology. Nowadays this surgical program consists of a total cavopulmonary connection (TCPC), by anastomosing systemic veins to the pulmonary arteries (PAs), excluding the right-sided circulation from the heart. As a result of imaging, surgical, percutaneous, and critical care improvements, survival in this population has steadily increased. However, the Fontan physiology chronically increases systemic venous pressure causing systemic venous congestion and decreased cardiac output, exposing patients to the failure of the Fontan circulation (FC), which is associated with a wide variety of clinical complications such as liver disease, cyanosis, thromboembolism, protein-losing enteropathy (PLE), plastic bronchitis (PB), and renal dysfunction, ultimately resulting in an increased risk of exercise intolerance, arrhythmias, and premature death. The pathophysiology of the failing Fontan is complex and multifactorial; i.e., caused by the single ventricle dysfunction (diastolic/systolic failure, arrhythmias, AV valve regurgitation, etc.) or caused by the specific circulation (conduits, pulmonary vessels, etc.). The treatment is still challenging and may include multiple options and tools. Among the possible options, today, interventional catheterization is a reliable option, through which different procedures can target various failing elements of the FC. In this review, we aim to provide an overview of indications, techniques, and results of transcatheter options to treat cavopulmonary stenosis, collaterals, impaired lymphatic drainage, and the management of the fenestration, as well as to explore the recent advancements and clinical applications of transcatheter cavopulmonary connections, percutaneous valvular treatments, and to discuss the future perspectives of percutaneous therapies in the Fontan population.

## Introduction

It has been 50 years since Francis Fontan designed the operation that offered for a wide range of congenital cardiac lesions with a single ventricle (SV) physiology (1). Nowadays this surgical program consists of a total cavopulmonary connection (TCPC), by anastomosing systemic veins to the (pulmonary arteries) PAs, excluding the right-sided circulation from the heart. As a result of imaging, surgical, percutaneous, and critical care improvements, survival has steadily increased, leading to a currently observed 30-year survival rate up to 85% (2, 3). Indeed, the worldwide population of patients with a Fontan circulation (FC) grew to an estimated 50,000–70,000 patients in 2018, with 40% of patients > 18 years of age (4).

However, in the absence of a sub pulmonary ventricle, FC is characterized by chronically elevated systemic venous pressures and decreased cardiac output, exposing those patients to the failure of the Fontan circuit, which remains difficult to treat (5).

Failure of FC is associated with a wide variety of clinical complications, including acute and chronic circulation failure with congestion and ascites, cyanosis, exercise intolerance, arrhythmias, thromboembolism, protein-losing enteropathy (PLE), plastic bronchitis (PB), liver disease, renal dysfunction, and premature death (4–6).

Due to the complexity of the failing Fontan pathophysiology, as well as the multicausal aspect of clinical complications, the therapeutic armamentarium may include multiple options and tools. Among the possible options, and thanks to its recent exponential development, interventional catheterization has a place of choice today, through which different procedures can target each failing element of the FC. Indeed, various percutaneous cardiac interventions, more or less recent, might either treat a focused lesion or palliate a Fontan-related dysfunction, in order to avoid end-organ failing Fontan status.

In this review, we will (1) describe the indications, techniques, and results of the transcatheter approach for basic lesions such as cavopulmonary stenoses or collaterals occlusions, (2) highlight the endovascular management of the fenestration between the systemic venous return and the pulmonary venous atrium during patients’ course, which may be either closed or created, depending on patients’ conditions, (3) detail the basic principles and indications of catheter-based lymphatic interventions in patients with Fontan circulation, (4) explore the recent advancements and clinical applications of transcatheter cavopulmonary connections as well as (5) percutaneous valvular treatments and (6) discuss the future perspectives of percutaneous therapies in the specific population of patients with Fontan circulation.

## Transcatheter approaches to Fontan circulation basic lesions

### Conduit obstruction

From the first description in 1971 (1), many modifications to the Fontan operation have been reported with different conduit positions and materials. Lee et al. (7) described the best TCPC with lateral tunnel or extracardiac conduits (ECC). Recently, the use of lateral tunnel has been abandoned for the high incidence of arrhythmias and stenosis, with ECC being the most used conduit.

Several studies (8, 9) described a progressive reduction of the internal diameter of the conduits due to intimal lining and a study (7) has reported a 14–18% decrease in the internal conduit diameter during follow-up. Moreover, there is a subgroup of patients with Fontan who develop significant conduit stenosis (8).

Conduit stenosis determines a reduction of the energetic efficiency of the Fontan pathway, with consequent energy dissipation, impaired flow distribution, decreased diastolic filling, and limited ventricular pre-load reserve (9).

Clinical manifestations range from reduced exercise tolerance to Fontan failure.

**FIGURE 1:**
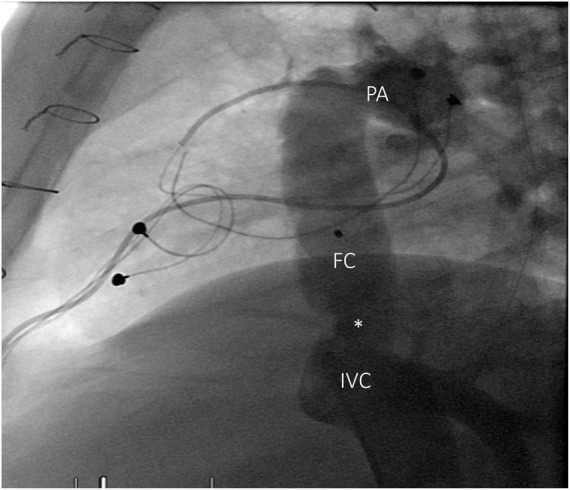
Angiography of inferior vena cava, lateral view. Stenosis at the proximal anastomosis (white asterisk) of a Fontan conduit, 15 years after intervention. FC, Fontan conduit; IVC, inferior vena cava; PA, pulmonary arteries.

Stenosis has been described at various sites of the conduit, with a discrete or diffuse appearance. Mechanisms of stenosis include the endothelialization process (mainly described in Dacron conduits), stretching related to physical growth, surgical scars, thrombus formation, and sometimes extrinsic compression as demonstrated by the presence of a typical scooped-out aspect of the left-sided or right-sided ECC (Figure 1).

Several geometric characteristics, such as inferior vena cava (IVC) diameter, right pulmonary artery (RPA) size, and discrepancy between the pulmonary arteries, seem to correlate with ECC stenosis, which can worsen significantly over time in the absence of percutaneous interventions (10). Echocardiography is rarely useful in the diagnosis, so a recently published scientific statement from the American Heart Association (11) recommended the use of cardiac magnetic resonance (CMR) or computed tomography (CT) in the follow-up of patients with Fontan.

Indications to treatment are not clear since a real gradient is not always present, but some authors advocate a reduction of the internal conduit diameter of 25% as hemodynamically relevant (10).

Actually, pressure gradients are irrelevant and only the conduit/vessel size matters. In patients with Fontan, normal size of the venous system and pulmonary arteries is crucial for flow optimization.

Catheter interventions are preferred for treating isolated obstructions. Various stents have been used for this purpose, with covered stents used only in the presence of highly calcified conduits. Pre-dilatation of the stenosis was recommended only in the study of Mets et al. (12) and it was used in 92% of patients with a lateral tunnel. Post-dilatation with a high-pressure balloon is advised in order to achieve an average stent final diameter superior to 16 mm/m^2^ (13).

Acute results of the procedure are very good with a success rate of 100% (10, 11, 14). Complication rate ranges from 0 to 10% and includes conduit dissection, well managed with covered stent implantation (10, 11). All the patients experienced an improvement in NYHA functional class and exercise capacity. At follow-up, successful re-dilatation of the conduit stent was described particularly in young patients with growth potential (12).

### Pulmonary arteries obstruction

One of the effects of FC is the introduction into the pulmonary bed of a non-pulsatile flow with low pressure. This non-physiological blood flow can negatively affect pulmonary artery growth.

Several studies (15, 16) have reported a decrease in indexed PAs diameters with time in patients with Fontan. The group of Restrepo et al. (17) quantified pulmonary vessel growth and flow rate changes with CMR in Fontan circuits. They found an increase in pulmonary flow rates proportional to the body surface area with no concomitant vessel diameter matching. These results translated into important energy dissipation in the conduit and the effect was particularly evident in the left pulmonary artery (LPA).

Moreover, a recently published study (18) showed that the pulmonary artery size expressed as Nakata index is an independent predictor for functional status expressed as pVO2 in patients with Fontan.

For these reasons, periodical evaluation of pulmonary arteries with CMR or CT is strongly recommended to guarantee a prompt treatment in the presence of stenotic or hypoplastic pulmonary segments. A percutaneous approach using either balloon angioplasty or stenting is the preferred method of treatment in these vulnerable and multioperated patients. On the one hand, a pulmonary angioplasty is usually indicated for the treatment of significant peripheral branch pulmonary artery stenosis in small patients in whom primary stent implantation is not an option. On the other hand, stent implantation is indicated for the treatment of significant proximal or distal branch pulmonary artery stenosis when the vessel/patient is large enough to accommodate a stent that is capable of being dilated to the adult diameter of that vessel (10). During stenting positioning, careful attention must be paid to peripheral branches in order not to alter an already damaged pulmonary bed and to bronchial compression, particularly in complex anatomies. Usually, non-covered stents are used in order to avoid any jailing of the surgical pathway or PA branches (19).

### Systemic to pulmonary venous collaterals

Systemic to pulmonary venous collaterals (SPVCs) are common findings in patients with Fontan and their etiology is still debated although the most assumed embryological structures “recanalize” due to the chronic elevation of central venous pressure (20).

SPVCs usually originate from IVC or superior vena cava (SVC), from the innominate vein, or from the azygos and hemiazygos system, and are in connection with pulmonary veins or common atrium. Large SPVCs often produce systemic arterial de-saturation with secondary erythrocytosis and can be a source of paradoxical embolism.

Percutaneous closure of SPVCs with coils or vascular plugs has been largely described mainly in form of case reports or small patients’ series. No procedural complications were described but clinical results are various. Some described a saturation improvement, particularly during the brief follow-up (21, 22) and others did not (23). Only a study from the Mayo Clinic group (21) analyzed the effect of SPVC embolization on mortality. They reported decreased 5-year survival of patients who underwent embolization compared with those who did not; in the multivariate analysis, SPVC embolization was the strongest risk factor for mortality. They recognized a threshold circuit pressure of 18 mmHg for embolization, since 50% of deaths after embolization occurred in patients with Fontan pressure > 18 mmHg. However, one should know that SPVC closure is not always performed as they naturally form as a “pop-off” under specific conditions and may reform again despite the closure.

### Aortopulmonary collateral arteries

Aortopulmonary collateral arteries (APCs) are common in patients with univentricular heart. They have been identified in up to 84% of patients undergoing catheterization in preparation for a Fontan procedure (24). They can be defined as vessels that pass from branches of the aorta to the pulmonary vascular bed. Some APCs represent vessels that pre-exist, while others consist of *de novo* vessels. The precise causes of APC formation are not well known. The most commonly suggested stimuli are cyanosis and decreased pulmonary flow volume, velocity, and pulsatility, typical of the Fontan circuit. APCs usually form in a lung that does not adequately participate in gas exchange in order to improve oxygenation, such as in PA branch stenosis.

APCs flow effect is a volume overload of the single ventricle with an increase in the atrial pressure and with a consequent increase in transpulmonary gradient and pulmonary pressure, detrimental for patients with Fontan. Moreover, APCs flow can compete with the systemic venous flow determining an energy loss in the Fontan circuit.

The real clinical significance of APCs and indications for the embolization are, however, not well defined.

Some authors advocate large APC s embolization prior to Fontan completion in order to reduce hospitalization days and pleural effusion duration after the operation (25), and others recognized APCs embolization as mandatory in the immediate period after Fontan completion, in the absence of anatomic defects, when signs of heart failure are evident or in the presence of long-lasting effusion.

**FIGURE 2:**
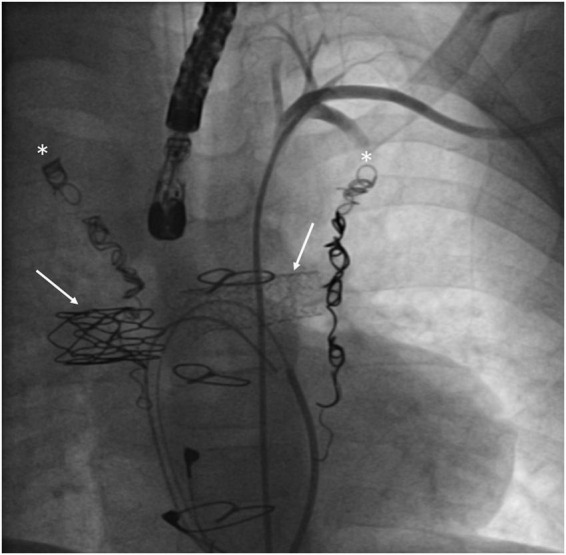
Patient with a Fontan failure who underwent coil embolization of both mam-mary arteries (white asterisks) and stenting of right and left pulmonary arteries (with arrows).

APCs embolization is usually performed with coils (Figure 2), and all the collateral vessels must be occluded from the distal to the proximal part in order to avoid recanalization and the procedure is at low risk.

### Aortic arch obstructions

In patients with Fontan, all potentially important hemodynamic derangements involving the systemic circulation such as significant atrioventricular valve regurgitation or aortic arch obstruction should be carefully evaluated for possible interventional approaches.

Transcatheter treatment of aortic arch obstruction in using balloon angioplasty has been well described but may result in inadequate relief of aortic shape with a non-negligible rate of recurrences. Stent implantation has been shown to provide excellent acute relief of aortic arch obstruction but long-term outcomes of this technique in patients with Fontan are necessary to study (26).

## Percutaneous management of conduit fenestration

**FIGURE 3:**
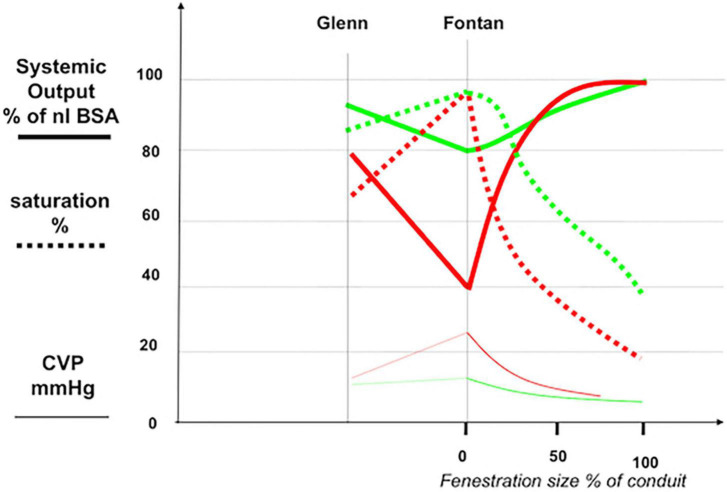
Effect of various degrees of pulmonary bypassing in a Fontan circuit on systemic output (thick line), saturation (dotted line), and systemic venous congestion (thin line). A “good Fontan” (green or lighter lines) with low neoportal resistance has a cardiac output (solid green line) of about 80% of normal for BSA, with high saturations (dotted green line) and a slightly raised CVP (thin green line). The “bad Fontan” (red lines) with a high neoportal resistance has comparable saturations (dotted red) but with a very low output (solid red) in the presence of a high CVP (thin red). Partial bypassing of the Fontan portal system by a fenestration consistently increases systemic output and lowers venous congestion but may give rise to clinically intolerable degrees of cyanosis (effects of fenestration size can be viewed at the bottom right of the graph). BSA: body surface area; CVP: central venous pressure. Adapted with permission from Gewillig et al. (28).

When creating an FC, the surgeon puts the pulmonary circulation like a dam upstream of the ventricle (27). A new critical bottleneck is then created with new physical forces regulating flow throughout the system. Like any dam, upstream congestion and downstream limited flow are enforced. However, excessive damming causes extreme congestion or very low output which is associated with unacceptable morbidity and mortality. In order to avoid such poor clinical conditions, the treating team may choose to go for partial damming, leaving a bypass between the systemic venous return and the ventricle. Thereby, at least in the early post-operative phase, episodes with extreme congestion or low output can be avoided. As the size of this bypass is critical, it is called fenestration. When a fenestration is too small, excessive congestion and low output will persist; when too large, cyanosis may be detrimental (28). A patient with good Fontan hemodynamics (broad connection, low pulmonary vascular resistance, low ventricular filling pressure) will not need a fenestration; when poor hemodynamics are present, the clinician may not find an acceptable balance between congestion/low output and cyanosis (Figure 3).

### Creating fenestration or other forms of residual shunting

#### Primary fenestration at the time of surgery

• One (or more) holes in the conduit: a well-defined opening (usually punch hole 4.0–5.0 mm) is made between the ICV conduit and the common atrium; the atrial wall is sutured a few millimeters away from the edge to avoid early spontaneous closure. In “high risk” patients, the team may choose to leave two holes at least 6 mm apart which allows sequential closure.
• Small Gore-Tex graft after bypass or in the early post-operative period (29)
• Modified Hraska or innominate vein turn-down procedure (30–32): this procedure leaves the thoracic duct at low atrial pressure: the innominate vein is connected to the atrium, and the left jugular vein (LJV) is sub-totally banded. Limited de-saturation will be caused by the venous flow from the left subclavian vein and the minimal flow through the banded LJV. This procedure is indicated when the patient has a high risk of lymphatic failure (previous chylothorax, pathological T2-weighted CMR scan).
• Kawashima procedure (33): in case of azygos continuation of the IVC, connecting the SCV to the PA results in the Kawashima procedure. The hepatic veins are not only left to drain directly to the (common) atrium causing some mild de-saturation but also predisposes the patient to the development of arteriovenous malformations after some years.
• Brizard procedure (34): the three major hepatic veins (right, middle, and left) are separated and connected to the common atrium. An extracardiac conduit connects the hepatic portion of the IVC to the PA. The other hepatic veins, including the vein draining the caudate lobe and multiple venules that normally drain into the IVC, are excluded by a covered stent in the IVC.

#### Secondary fenestration: Percutaneous creation

• Puncture between high-pressure venous system and systemic atrium. Different substrates can be perforated:

– Atrial septum in patients with atriopulmonary connection (35)
– The conduit to the common atrium;
– The thoracic IVC stump to the common atrium (36)
– The right pulmonary artery to the systemic atrium (37)

The puncture can be performed with a Brockenbrough needle or with radiofrequency (will not perforate Gore-Tex) from femoral, jugular, or hepatic access. Except for the real atrial septum, all puncture sites involve going through the pericardial space but tamponade is extremely rare as most patients have already several sternotomies, with multiple adhesions that limit blood accumulation with systemic venous pressure; after perforating, the atrial wall prolonged patency of the fenestration is usually obtained by a stent or fenestrated occluder implantation (37, 38).

• Spontaneous veno-venous collaterals may form with time. Similarly, a minute persisting left SVC may “grow” and allow venous blood to flow from the coronary sinus to the atrium.
• the modified Hraska can be performed as a secondary procedure, either surgically or in selected patients percutaneously by connecting the innominate vein to the atrium or atrial appendage (39).
• percutaneous Fontan take-down: by expanding a covered stent from the ICV through a fenestration into the atrium, the Fontan circuit is taken down and converted into a partial cavopulmonary circulation.

### Fenestration closure

Persistence of fenestration can result in cyanosis both at rest and increasingly during exercise, resulting in limited exercise tolerance (40), with a continuous risk for a paradoxical embolus. All these side effects can be abolished by closing the fenestration, but at the expense of increased congestion and lower output.

The choice for closure should be tailored to the patient as it is choosing between quality and quantity: a fenestration with less congestion and more output will predispose to longer life, but the cyanosis may cause less quality.

When considering fenestration closure, patient selection prior to the eventual procedure is crucial: the better candidate is typically the better Fontan patient with no post-operative hydro- nor chylothorax, no need for diuretics nor pulmonary vasodilators, no persisting hepatomegaly, no ascites, and an active lifestyle. Fontan fenestration closure may not be advisable in cases with a high baseline left atrial pressure or a significant increase in Fontan pressure on balloon occlusion testing (41).

**FIGURE 4:**
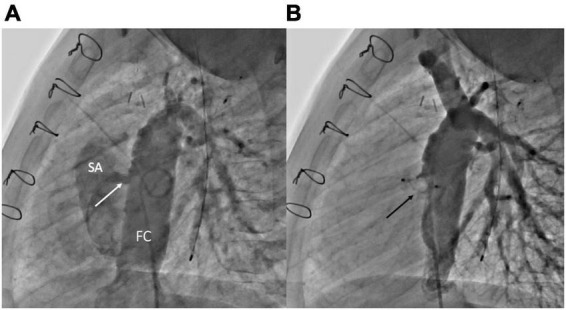
Fenestration closure using vascular plugs. **(A)** Lateral angiogram of a Fontan conduit showing a fenestration (white arrow) between the conduit and the single atrium. **(B)** Lateral angiogram following fenestration closure using an Amplatzer Vascular Plug II (Abbott) (black arrow). FC, Fontan conduit; SA, single atrium.

**FIGURE 5:**
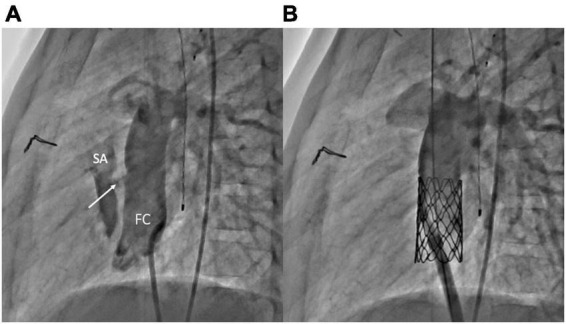
Fenestration closure using covered stents. **(A)** Lateral angiogram of a Fontan conduit showing a fenestration (white arrow) between the conduit and the single atrium. **(B)** Lateral angiogram following fenestration closure using a covered CP stent (Numed) implantation within the conduit. FC, Fontan conduit; SA, single atrium.

A fenestration can be closed using several devices and approaches:

1. Initially, atrial septal defect occluders were used, but these required a large deployment system that is bulky and expensive (42).
2. Currently, most interventionists use thin patent ductus arteriosus devices like Amplatzer duct occluder II or vascular plugs that are versatile, easily delivered through a small sheath, relatively cheap, and easily retractable even after a few days (Figure 4) (43, 44). The low-profile devices developed for para-valvular leak closure may also be used for this purpose (45).

3. The placement of the covered stent within the conduit may exclude the fenestration and also allows the reopening of a compressed or stenotic conduit (Figure 5) (46–48).

### Fenestration manipulation

An existing fenestration can be enlarged or restricted. Enlargement with a simple balloon dilation yields usually no lasting effect, and most interventionalists will prefer to use a stent (straight or in diabolo) (49); depending on the indication, the fenestration will be enlarged until saturation of 80–85% or lower is obtained.

The fenestration can also be percutaneously restricted without being closed using several techniques including (1) insertion of multiple or long-covered stents in the existing fenestration stent, (2) partial collapse of a preexisting stent with a snare (50), (3) implantation of an atrial flow regulator device (37, 51), or (4) close the second of two fenestrations.

## Lymphatic interventions in patients with Fontan

The lymphatic system has been overlooked for years mainly owing to difficulties to reach and image this system. Twenty years ago, Cope demonstrated that the thoracic duct (TD) could be reached after pedal lymphangiography and that the embolization of the duct with coils was an effective treatment of chylothorax from various etiologies (52). Ten years later, Telinius demonstrated that the lymphatic system was complex, innervated and that it was dependent on pre- and after-load (53). In the meantime, Itkin and Dori in Philadelphia developed new techniques to visualize the lymphatic system opening the door to new therapeutic strategies (54, 55). They developed dynamic contrast-enhanced magnetic resonance (MR) lymphangiogram and T2-weighted MR to visualize the central lymphatic system and to characterize lymphatic abnormalities and other modalities such as liver lymphangiography.

The effect of the FC on the lymphatic system and the role of the lymphatic system in Fontan complications are not fully understood yet, but it is now well established that lymphatics play a key role in Fontan complications and failure (28, 56, 57). The Fontan physiology chronically increases systemic venous pressure causing systemic venous congestion, which impedes effective drainage of the lymphatic system. Furthermore, increased systemic venous pressure causes significant increases in lymph production, mainly from the liver (58). The double burden of increased lymph production in the system, in which drainage is already impeded by higher systemic venous pressure, is likely one of the main mechanisms of lymphatic dysfunction. The net effect of the impaired lymphatic drainage and increased lymphatic pressures is over-distention and rupture of lacteals with leakage resulting in retrograde lymphatic flow into low-pressure lumens, e.g., gut and bronchi (55–57). What remains unknown is why some patients will develop complications, while many others will remain asymptomatic suggesting more subtle mechanisms for the development of these diseases like local abnormalities of the lymphatic system. Two classical complications of the Fontan circulation are directly linked to the lymphatic system, PLE and PB. They are a major cause of attrition in the Fontan population with an incidence of 3–20% for PLE and 4–14% for PB (59). Medical treatments are disappointing. Hraska demonstrated the major role of elevated pressure in the lymphatic in the PLE by decompressing the TD by diverting its drainage into the systemic atrium (connecting the innominate vein to the atrium) at the expense of a mild de-saturation and showed PLE resolution (30).

**FIGURE 6:**
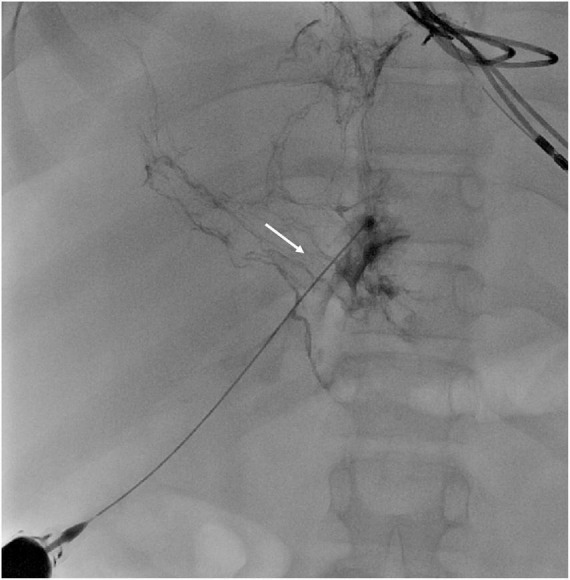
Liver lymphangiography in a Fontan patient with protein losing enteropathy: note the dilated lymphatics in the peri-portal area draining toward the gut (white arrow).

**FIGURE 7:**
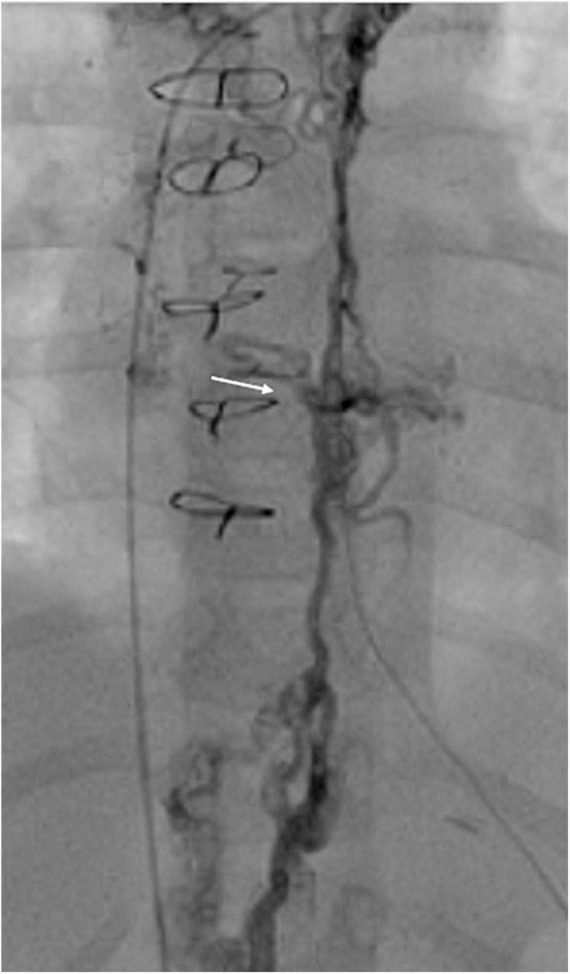
Thoracic duct lymphography in a Fontan patient with plastic bronchitis: note the dilated thoracic duct with multiple and bilateral leaks toward the lungs (white arrow).

Ikin and Dori demonstrated abnormal anatomy or function of the lymphatic system in the gut for the PLE and of the TD in the PB by MR-lymphography and by selective opacification of the lymphatics. They demonstrated hepatic lymph leakage into the duodenum *via* hepatoduodenal communications by injecting dye into the peri-portal lymphatics and observing dye leakage into the duodenum using endoscopy. They showed the same abnormalities in the trachea by injecting dye in the TD and demonstrating leakage of the dye into the trachea with bronchoscopy. Percutaneous obliteration of these lymphatic fistulae resulted in the reduction of lymph leakage to subclinical levels, bringing this technique as an additional treatment option for PLE and PB (54, 55, 60, 61). An example of liver lymphangiography in a Fontan patient with PLE and PB is shown in Figures 6, 7, respectively.

Itkin’s team and others have reported small series of patients with PLE, treated by liver lymphangiography followed by liver lymphatic embolization with a combination of lipiodol and glue, resulting in PLE resolution (60, 62). Using ultrasound guidance, a 22-gauge Chiba needle was inserted percutaneously in the liver parenchyma up to the peri-portal space. The desired needle position for embolization treatment was reached when iodinated contrast agent injection demonstrated the hepatoduodenal lymphatic vessels with run-off toward or even into the gut. In patients with Fontan, these lymphatics are typically dilated. Embolization of the lymphatics was performed with a 1:4 mixture of n-butyl-2-cyanoacrylate NBCA (Histoacryl^®^, B. Braun, Barcelona, Spain) and 1.5 mL Lipiodol^®^ (Guerbet, France). Several punctures were performed in order to embolize lymphatics from the right and left liver. In a retrospective series, Maleux et al. reported significant improvement in the quality of life and normalization of albumin levels in six of seven patients after peri-portal lymphatic embolization after limited follow-up (61).

Dori et al. reported on a series of patients with PB. Intranodal lymphangiogram was performed to opacify central lymphatic vessels (large lumbar lymphatics vessel or cisterna chyli). The target vessel was accessed *via* transabdominal puncture using a Chiba needle. A microwire was then advanced into the TD followed by a microcatheter. Four embolization techniques were used according to the anatomies encountered: (1) embolization of the TD with coils and glue; (2) embolization of the TD with lipiodol; (3) selective embolization of TD abnormal branches with glue and lipiodiol; and (4) isolation of TD abnormal branches by stenting of the TD with a covered stent. In this series of 18 patients, 17 were embolized and 15 had significant improvement of the symptoms with a mean follow-up just shorter than 1 year (62).

Although promising, these treatments do not address the cause of the lymphatic disorder. Thus, the optimization of the Fontan circuit prior to the lymphatic intervention is paramount. Likewise, supportive treatment such as pulmonary vasodilators in PB must be maintained after embolization.

The lymphatic system is now recognized as a major player in FC. As knowledge increases on this overlooked circulation, new treatments for failing Fontan or even preventive strategies based on the evaluation of the fragile system are likely to emerge.

## Percutaneous Fontan completion

Despite the Fontan procedure has undergone modifications over the last several decades in an attempt to improve patients’ outcomes, it is still associated with long-term complications. With the development of interventional cardiology, some interventionists along with surgeons have developed innovative hybrid strategies to perform transcatheter Fontan completion *de facto* by decreasing the number of these surgical interventions to palliate patients with single ventricles. These techniques involve a surgical preparation (“surgical pre-conditioning”) at the time of the bidirectional cavopulmonary connection (BCPC) to set the patient up for percutaneous transcatheter completion at a later stage (63–66).

Following experimental deployment in a few animals, a group in Berlin conducted initial utilization in humans between 1994 and 1995 (63, 67, 68) when they fashioned multiple fenestrations into the baffle of a lateral tunnel Fontan in 18 high-risk patients. SVC was banded above the cavoatrial junction to direct the SVC blood flow primarily to the pulmonary vasculature. The venous return from the IVC enters the systemic atrium through the fenestrated baffle, accomplishing a bidirectional Glenn circulation. At the subsequent transcatheter stage, the fenestrations were occluded using devices and the cardiac end of the SVC was reopened widely by simple balloon dilatation. A similar approach was reported in 2007 by Sallehuddin and colleagues. and 2011 in 34 patients (69, 70). They fashioned an intra-atrial lateral tunnel with a large 10–14 mm fenestration to create free communication between the tunnel and the atrium. The cardiac end of the SVC was then patched to maintain the physiology of BCPC. At the time of the transcatheter completion, the SVC patch was perforated using radiofrequency (RF) energy, balloon-dilated, and then stented. The aperture was then closed with a device (or covered stent) leading to the completion of the FC.

In all instances, these “non-surgical” completions result in lower use of inotropes, reduced effusion rates, and shorter lengths of intensive care unit and hospital stay (65, 67, 69, 70). Despite interesting clinical results, dissemination of the technique remained limited. But, multiple teams continued to work with animals to try to improve the surgical pre-conditioning.

**FIGURE 8:**
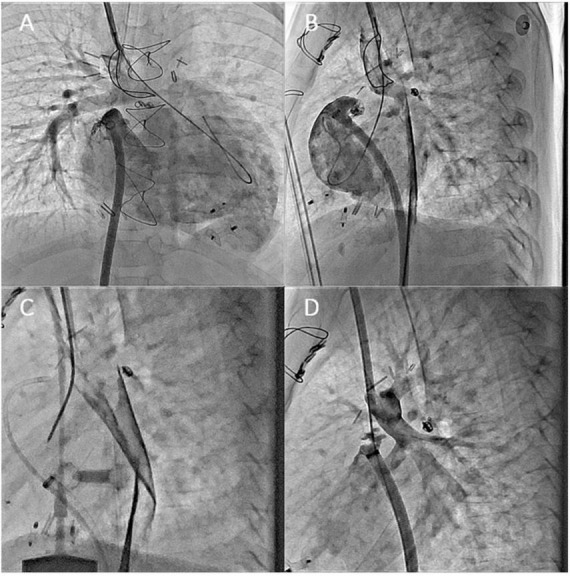
Angiographic images from 5-years-old boy who underwent transcatheter Fontan completion. **(A,B)** Simultaneous frontal and lateral injections of the right atrium and Glenn showing the distance between the two circuits. Please notice the clips at SVC and IVC levels; **(C)** lateral view of the connection created with a needle between Glenn and RA; **(D)** lateral angiogram after perforation showing no significant bleeding and SVC to RA connection.

**FIGURE 9:**
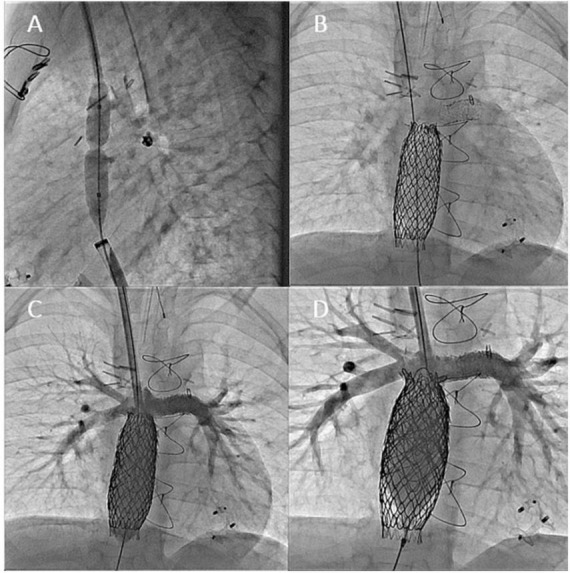
**(A)** Ballooning of the neo-connection to help the advancement of IVC sheath to the Glenn; **(B–D)** angiograms done after the completion of the intracardiac Fontan with covered stents. IVC flow is now draining from IVC to PAs without restriction. The for-ward flow (RV to PA) was occluded by positing a covered stent from RPA to LPA.

In 2000, Klima et al. from Hannover successfully combined a unidirectional cavopulmonary anastomosis without cardiopulmonary bypass with percutaneous implantation of the self-expanding stent graft in 10 sheep to complete an FC (71). Nonetheless, this non-survival animal model excludes reproduction in human subjects. A similar technique was reported in 2004, wherein a BCPC was constructed together with suturing of the caudal end of the SVC to the closed under the surface of the RPA. The authors implanted radio-opaque markers at the SVC and the IVC for subsequent localization and completed the FC percutaneously by the deployment of an appropriately sized covered stent from the IVC to the RPA (65). This technique does, however, pose concerns regarding stent stability and stent extension into the RPA, as well as a potential exclusion of a hepatic vein or leaks around a stent; both can result in the right to left shunt and significant de-saturation. There are also the risks of occluding the hepatic veins and the possibility of stent migration. In 2005, Konstantinov et al. modified this technique in a non-survival animal model by placing stents around the cardiac stems of the SVC and around the IVC at the level of the diaphragm (72, 73). Apart from eliminating stent migration, the external stents also serve as radiological guides during the completion procedure. The stent around the IVC diminishes the risks of leakage of hepatic venous blood into the systemic atrium. It also provides guidance for an accurate stent deployment that circumvents hepatic vein occlusion. A similar approach was described by Metton et al. (74) and Gerelli et al. (75). All the offered approaches result in the creation of an intra-atrial tunnel. An example of transcatheter Fontan completion is shown in Figures 8, 9.

The next challenge was to fabricate an extracardiac-type Fontan circuit with minimal obstruction to the cavopulmonary pathway and possibly dilatable to match the growth of the patient. The attributes of ideal surgical preparation for the transcatheter completion of FC include (1) achievement of a safe and viable preparation with limited use of cardiopulmonary bypass and avoidance of ischemic arrest; (2) a minimal quantity of foreign material left in the systemic atrium; (3) maintenance of normal sinus rhythm; (4) limited atrial scarring; (5) the use of conduits of sufficient diameter to accommodate future cardiac growth and thus avoid surgical redo; and (6) most importantly, safe completion of FC using transcatheter techniques.

With these attributes in mind, Boudjemline et al. designed, in 2013, a circuit that mimics the actual surgical technique of extracardiac to allow for transcatheter completion (76, 77). First, they integrated a rim of right atrial tissue to facilitate the implantation of an adult-size conduit (16–22 mm) in an end-to-end fashion to the IVC. A large fenestration allowed free flow from the IVC to the right atrium. This also served to provide a complete washout of the conduit to prevent stasis and reduce the possibility of thrombus formation. They then interposed a short segment of natural expendable tissue between the Gore-Tex conduit and the SVC to address the discrepancy between their diameters. Animals included in the study had a successful conversion to an extracardiac circuit by reopening the SVC-conduit connection and closing the large fenestration by placing a long-covered stent. The creation of an extracardiac Fontan was reported in humans using a similar approach in 2017 and 2020. Reports in the literature involve, however, small numbers of patients with limited follow-up intervals (78, 79).

The collaboration between surgeons and interventional cardiologists is remarkable, providing an environment to successfully deal with challenges. Surgical pre-conditioning and transcatheter Fontan completion are now at the next level; the strategy is in our opinion ready for dissemination. Future clinical studies with a larger population are of course needed to demonstrate the efficacy and safety of this new approach. Results should at least match with the results of the conventional care of patients with single ventricle (80).

## Transcatheter atrioventricular valve repair

**FIGURE 10:**
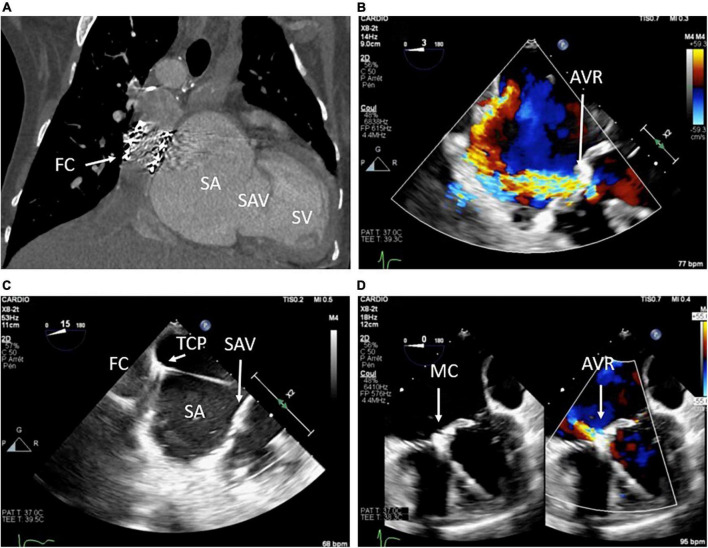
Percutaneous atrioventricular valve edge-to-edge repair procedure in a patient with Fontan Circulation. **(A)** Computed tomography scanner showing the Fontan conduct (FC) connecting the inferior vena cava to the pulmonary artery, the single atrium (SA), the single atrioventricular valve (SAV), and the single ventricle (SV); **(B)** transesophageal echography with color Doppler showing the high-grade regurgitation of the atrioventricular valve (AVR); **(C)** echo guided puncture of the Fontan conduct connecting the inferior vena cava and the left pulmonary artery; **(D)** result after Mitra-clip (MC) implantation responsible for a dramatic decrease of the AVR.

Right atrium pressure elevation is inherent to FC and depends on pulmonary vascular resistances, single ventricle systolic and diastolic functions, and atrioventricular valve continence. In adults with FC, the appearance of atrioventricular valve regurgitation (AVR) is common and sometimes responsible for an increase in left atrium pressure. When the impact of AVR is significant, surgery could be required but the perioperative risk is usually high or prohibitive. Because in adults with degenerative mitral or tricuspid regurgitation, percutaneous edge-to-edge repair (PETER) has proved its efficacy and safety to treat regurgitation, the idea to extend ETER to the atrioventricular valve in an adult with congenital heart disease (ACHD) logically made its way. In case of FC, reaching the systemic atrium is challenging and could require the Fontan conduit puncture or direct surgical access to the atrium *via* thoracotomy. Nevertheless, if the access is feasible and the orientation of the MitraClip is suitable, ETER of the AVR could be efficient. In patients with ACHD, only a few cases of ETER have been published, only to treat systemic tricuspid valve regurgitation, but none in case of FC (81, 82). In the in-press French multicentric study including cases of ACHD treated by ETER, we report four cases of patients with FC. The MitraClip device (Abbott, Santa Clara, CA) was used in all cases. This native technic seems to be feasible, efficient, and safe, and the results are encouraging. An example of percutaneous atrioventricular valve edge-to-edge repair performed in a patient with Fontan circulation is shown in Figure 10.

## Transcatheter valvulation of the cavopulmonary conduit

When focusing on the infradiaphragmatic venous return pattern of patients with Fontan, it has been shown that forward flow into the pulmonary circulation is significantly reduced in the upright position because of gravity (83).

Hence, several surgical valved conduits such as homografts or Contegra have been considered to achieve the cavopulmonary connection and palliate this limitation.

Indeed, as a part of the IVC blood backflows during expiration/upright position, putting a valve between the IVC and the PAs would theoretically increase single ventricle pre-load and cardiac output and decrease subdiaphragmatic complications. These hypotheses have been further supported by preclinical experiments (84).

For instance, Santhanakrishnan et al. reported an *in vitro* experimental setup consisting of an FC with a bovine venous valve inserted in the IVC, below the TCPC conduit. In this setting, the authors showed that (1) valve closure was effective and occurred for 15–20% of the total cardiac cycle, (2) the hepatic venous pressure decreased by 5–10 mmHg, and (3) the energy loss through the TCPC was lowered to 20–50% of baseline.

Unfortunately, these theoretical benefits were not confirmed in clinical practice. Indeed, the reported results with valved conduits implanted during the last surgical stage of the Fontan program were disappointing, mostly related to early stenotic degeneration of the substrate (85, 86).

In 2014, Malekzadeh-Milani et al. reported data of four patients with a failing Fontan who underwent percutaneous implantation of a Melody valve (Medtronic, Minneapolis, MN, United States) between the IVC and the PAs (87).

The Melody transcatheter valve, a bovine jugular vein sutured onto a platinum and iridium stent, was considered an ideal candidate for FC valvulation. Indeed, the bovine jugular vein has low closing pressure and is usually functioning with a slow and non-pulsatile flow. Moreover, the valve diameter (18–22 mm) is similar to that of the cavopulmonary surgical conduits (88).

Finally, its implantation from the femoral vein in a Fontan conduit is straightforward and technically simple, making the procedure to be safe.

In the Malekzadeh-Milani et al. series, two patients had severe and refractory PLE; one had severe edema and ascites; and one had very severe lower limb venous insufficiency. The Melody valve was implanted in all patients without complications. At follow-up, no acute or mid-term thrombosis was noted. Two patients remained stable; one had an improved condition with the disappearance of PLE. One patient died 3 months after valvulation after initial improvement; the cause was unrelated to the procedure.

**FIGURE 11:**
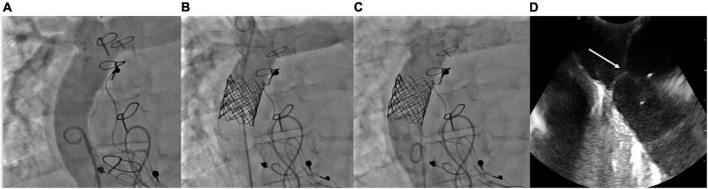
Melody valve implantation in a patient with a Fontan circulation. **(A)** Basal angiogram of the extracardiac conduit; **(B)** angiogram showing a competent Melody valve; **(C)** angiogram showing good forward flow between inferior vena cava and pulmonary arteries; **(D)** intra-cardiac echography 1 after implantation, showing competent Melody valve and perfect leaflets coaptation (white arrow).

These encouraging results emphasize the need for multicenter and prospective registries to (1) confirm the mid to long-term efficacy of this intervention and (2) better define the indications, the optimal timing, and the best candidates for this technique. Nevertheless, regarding the ease and the safety of Fontan valvulation, the latter may be at least considered as an option in failing patients with Fontan, as a bridge to transplant. A case of Melody valve implantation in a patient with failure Fontan is shown in Figure 11.

## Discussion—Perspectives

Even though it sounds a commonplace, the most important point to prevent complications in SV physiology is probably to obtain the most performant circuit at the time of Fontan completion. However, despite significant improvements that have been done over the years, perfection has not been reached yet.

What is the limiting factor in the FC is still under debate: indeed, when confronting with a patient with congestion and low cardiac output, potentially combined with a large thickened hypo contractile ventricle, the cardiologist’s reflector will point to the ventricle as the cause of all evil. However, current surgical and percutaneous techniques have highlighted that cavopulmonary repair is an extra-cardiac operation, where a new critical bottleneck is created upstream of the ventricle causing congestion and low output, as well as many secondary changes in ventricular, vascular, and lymphatic levels (27). Thus, to consider the problems in the FC only as a matter of ventricular function is extremely limiting.

The early and late goal in the FC is to maintain the best possible pulmonary vasculature with good capacitance, low resistance, and impedance, combined with a ventricle with the lowest possible filling pressures. Thus, pressure and kinetic losses must be minimized, and conduits and pulmonary artery stenosis are promptly treated (10, 18).

Some progresses in the knowledge of the role of the lymphatic system have been done in the last years. Most of the scientific efforts have been focusing on treating the lymphatic lesions when the FC fails. However, identifying patients with poor lymphatics resistance before Fontan completion or performing preventive maneuvers such as the decompression of the TD (30) in all patients might represent valuable approaches.

In theory, the optimal solution for the FC is to provide a sub-pulmonary pump, as nature has provided in mammals with a right ventricle; unfortunately, it is unlikely that medicine will outbreak the solution of millions of years of evolution. In this regard, the Fontan valvulation, increasing single ventricle pre-load and decreasing sub-diaphragmatic complications, might be a promising technique in future to come.

Lastly, considering that AVR is very common in patients with FC and that the advances in percutaneous treatments of AVR are rapidly growing, a good percutaneous option will certainly be welcomed to improve hemodynamics in patients with Fontan.

## Conclusion

Percutaneous interventions have been rapidly evolving over the last years and nowadays different procedures can target distinct failing elements of the FC. In next future, conjointly with a better understanding of the pathophysiology of the FC, the range of transcatheter procedures is expected to increase, hopefully contributing even more to improving patients’ clinical outcomes.

## Author contributions

ZJ conceived and designed the plan of the manuscript. All authors drafted one or two subsections of the manuscript, revised the manuscript critically, and agreed to be accountable for the content of the submitted work.
